# Supplementary material for: Complexome profiling of the Chlamydomonas psb28 mutant reveals TEF5 as an early PSII assembly factor
Source: Plant Cell. 2025 Mar 17;37(6):koaf055. doi: 10.1093/plcell/koaf055 (PMC12164590; doi:10.1093/plcell/koaf055)
Supplement: koaf055_Supplementary_Data [file koaf055_supplementary_data.zip › psb28_interactive.zip!/plots/intensity/qualityPlots/peptides/_Cre12.g545177.t1.1.html]

### Cre12.g545177.t1.1

Trivial name:   
SeqCoverage [median]: 12.60  
Mass: 29.88 kDa  
  
 

| Sequence | MissedCleavage | Mass [Da] | wtIntensity | muIntensity | Score |
| --- | --- | --- | --- | --- | --- |
| ASPGLLEAVSVEAR | 0 | 1397.75 | 462.53 | 0.00 | 73.85 |
| LAAQEAEAAR | 0 | 1028.53 | 232.79 | 492.81 | 104.20 |
| MEQQVQALTDK | 0 | 1289.63 | 0.00 | 329.09 | 87.30 |
|  |  |  | 695.3 | 821.9 |  |
